# Correlation of Lp(a), ApoB and oxLDL with Endothelial Damage Reading in Patients with Different Degrees of Coronary Atherosclerosis

**DOI:** 10.3390/ijms27031160

**Published:** 2026-01-23

**Authors:** Agnė Liuizė (Abramavičiūtė), Jolanta Laukaitienė, Renata Paukštaitienė, Viltė Marija Gintauskienė, Aušra Mongirdienė

**Affiliations:** 1 Department of Biochemistry, Lithuanian University of Health Sciences, 44307 Kaunas, Lithuania; agne.liuize@stud.lsmu.lt (A.L.); jolanta.laukaitiene@lsmu.lt (J.L.); 2 Department of Physics, Mathematics, and Biophysics, Medical Academy, Lithuanian University of Health Sciences, 44307 Kaunas, Lithuania; renata.paukstaitiene@lsmu.lt; 3 Department of Imunnology and Allergology, Medical Academy, Lithuanian University of Health Sciences, 44307 Kaunas, Lithuania; vilte.gintauskiene@lsmu.lt

**Keywords:** atherosclerosis, oxidized low-density lipoprotein cholesterol, vimentin

## Abstract

This pilot hypothesis-generating study evaluated whether lipid-related biomarkers (Lp(a), ApoB, and oxLDL), endothelial injury markers (endocan, vimentin), and extracellular matrix glycoproteins (TSP-1, TSP-2) reflect the severity of coronary artery disease (CAD) in patients with stable angina pectoris. 93 patients underwent invasive coronary angiography/coronary CT angiography. CAD severity was evaluated using Gensini, SIS, SSS, and CAD-RADS scores. CAD was confirmed in 76.3% (*n* = 71). OxLDL correlated with Gensini (r = 0.455; *p* = 0.006), atherosclerotic segments (r = 0.469; *p* = 0.005), arteries (r = 0.479; *p* = 0.004), revascularization indication (r = 0.318; *p* = 0.003), circumflex artery stenosis (r = 0.323; *p* = 0.005). OxLDL also correlated with vimentin (r = 0.459; *p* < 0.001). Vimentin correlated with Gensini (r = 0.480; *p* = 0.005), SIS (r = 0.349; *p* = 0.003), SSS (r = 0.320; *p* = 0.008), CAD-RADS (r = 0.331; *p* = 0.005), atherosclerotic segments (r = 0.515; *p* = 0.003), arteries (r = 0.384; *p* = 0.030), revascularization indication (r = 0.324; *p* = 0.003). Endocan, TSP-1, and TSP-2 showed no significant associations. These exploratory findings suggest that oxLDL and vimentin may be associated with CAD severity; however, confirmation in larger, prospective cohorts is required.

## 1. Introduction

Atherosclerotic cardiovascular disease remains the leading cause of morbidity and mortality worldwide, emphasizing the need for early detection of high-risk individuals and improved risk stratification beyond traditional lipid measurements.

Elevated concentrations of lipid-related biomarkers (lipoprotein(a) (Lp(a)), apolipoprotein B (ApoB), oxidized low-density lipoprotein (oxLDL), and markers of endothelial injury (endocan, vimentin), and extracellular matrix (ECM) glycoproteins (thrombospondin-1 (TSP-1), thrombospondin-2 (TSP-2) may indicate subclinical vascular damage, enabling earlier preventive and therapeutic interventions. OxLDL, Lp(a), and ApoB are closely involved in the pathogenesis of atherosclerosis, and endothelial responses to dysregulated lipoprotein metabolism, oxLDL, and increased reactive oxygen species (ROS) production play a pivotal role in the initiation and progression of atherogenesis [1,2,3].

Oxidative stress is a major risk factor contributing to the onset and progression of atherosclerosis. ROS promote inflammation, endothelial dysfunction, and altered lipid metabolism in early atherogenesis [1,4]. During oxidative stress, low-density lipoprotein cholesterol undergoes oxidative modification to oxLDL. OxLDL binds macrophage scavenger receptors (CD36, SR-AI/II, SR-BI), facilitating intracellular uptake. Accumulation of oxLDL within macrophages drives foam cell formation—a hallmark of early atherosclerotic lesions—and promotes both plaque progression and vascular inflammation [2,3,5].

Lp(a) and ApoB may help identify individuals at increased cardiovascular risk, even in the absence of traditional risk factors [6,7]. Lp(a) is a unique low-density lipoprotein (LDL)-like particle composed of lipids and a single ApoB-100 molecule covalently bound to apolipoprotein(a) (Apo(a). Apo(a) size is inversely proportional to circulating Lp(a) levels. Importantly, Apo(a) binds oxidized phospholipids, contributing to the pro-inflammatory and pro-atherogenic properties of Lp(a) [8,9]. Elevated Lp(a) is a strong genetically determined risk factor for atherosclerotic cardiovascular disease and promotes atherogenesis, thrombosis, and inflammation [8,10,11,12].

ApoB is a key prognostic biomarker present in all atherogenic lipoproteins except high-density lipoproteins and serves as an indicator of the total number of circulating atherogenic lipoprotein particles. It has two isoforms, ApoB48 (in chylomicrons/remnants) and ApoB100 (in very-low-density lipoprotein (VLDL), intermediate-density lipoprotein (IDL), LDL, and Lp(a)), which facilitate lipid transport and cellular cholesterol uptake via LDL receptors [7,13]. Elevated ApoB results from dietary factors, impaired clearance, or ApoB gene variants that reduce receptor binding, ultimately leading to increased LDL accumulation [7,14].

Endocan, an emerging biomarker of endothelial activation, is expressed by activated endothelial cells [15,16]. Endothelial dysfunction is widely recognized as an early event in atherosclerosis. Endocan exerts pro-inflammatory effects, supports vascular smooth muscle cell proliferation and migration, and contributes to neointima formation [16,17,18,19].

Vimentin, another marker of endothelial injury, correlates with circulating oxLDL levels and the presence of coronary artery disease (CAD) [20]. Vimentin participates in cellular migration, adhesion, division, and endocytosis and is expressed in endothelial cells, fibroblasts, macrophages, and other cell types [21]. Its expression increases during macrophage transformation into foam cells, underscoring its role in regulating macrophage infiltration during atherogenesis [22].

TSP-1 and TSP-2 are ECM glycoproteins increasingly studied for their involvement in atherosclerosis. TSP-1 is expressed by all vascular cell types and contributes to tissue repair, hemostasis, cell adhesion, migration, proliferation, ECM organization, and regulation of growth factor activity. It also inhibits nitric oxide-mediated endothelial angiogenic activity and platelet anti-thrombotic responses [23,24]. In contrast, evidence linking TSP-2 to CAD remains limited. Some studies report low or absent TSP-2 expression in atherosclerotic plaques [25], whereas others suggest that TSP-2 influences plaque stability by modulating matrix metalloproteinase-2 activity, thereby potentially reducing inflammation and maintaining ECM integrity [26].

Given their biological roles, these biomarkers may be involved in the development and severity of atherosclerosis. However, few studies have simultaneously examined lipid-related biomarkers, endothelial injury markers, and ECM glycoproteins in the context of CAD severity.

We hypothesized that higher levels of lipid-related biomarkers, endothelial injury markers, and ECM glycoproteins are independently associated with greater severity of coronary atherosclerosis. Therefore, this study aimed to investigate the relationship between lipid-related biomarkers Lp(a), ApoB oxLDL, and markers of endothelial damage (endocan and vimentin), and ECM glycoproteins (TSP-1 and TSP-2), and to evaluate the potential of these markers to reflect the severity of coronary atherosclerosis in patients with stable angina pectoris (SAP).

## 2. Results

After excluding ineligible patients, a total of 93 patients were included in the study. The median age was 57 years (range: 18–80 years), and the distribution of males and females was similar (45 (48.4%) males vs. 48 (51.6%) females).

For the assessment of coronary atherosclerosis, most patients underwent coronary computed tomography angiography (CCTA) (*n* = 78, 83.9%), while 35 patients (37.6%) underwent invasive coronary angiography (ICA); 21 patients (22.6%) received both examinations. Coronary atherosclerosis was identified in 71 patients (76.3%), and 17.4% required revascularization (percutaneous coronary intervention (PCI), *n* = 15 (16.1%); coronary artery bypass grafting (CABG), *n* = 2 (2.2%).

### 2.1. Baseline Characteristics of the Study Groups and Medication Use

The main characteristics of the subjects according to CAD status are presented in Table 1, and the medications taken at the time of admission are summarized in Table 2. Compared to patients without CAD (Non-CAD group), those with CAD were significantly older (*p* = 0.039), had a higher body mass index (*p* = 0.028) and systolic blood pressure (*p* = 0.026), and were more likely to have dyslipidemia (*p* = 0.002) and a positive family history of cardiovascular disease (*p* = 0.018) (Table 1). Other baseline characteristics, including abnormal intima–media thickness (IMT > 0.9 mm), elevated cardio-ankle vascular index (CAVI > 9.0), increased pulse wave velocity (PWV > 10 m/s), and suspected peripheral artery disease (PAD, defined as ankle–brachial index (ABI) < 0.9), did not differ significantly between the CAD and non-CAD groups (Table 1). There were no statistically significant differences in medication use between these two groups (Table 2).

Baseline characteristics and medication use among patients who underwent ICA were stratified by Gensini score. The corresponding data are summarized in Table 3 and Table 4. No significant differences were observed, except that PAD was more commonly suspected in patients with moderate CAD (Group 2) compared to all other groups. Additionally, patients with minimal and moderate CAD (Groups 1 and 2) were more likely to have arterial hypertension than those without CAD (Group 4); *p* = 0.03 (Table 3). No statistically significant differences in medication use were observed between the two groups (Table 4).

Baseline characteristics and medication use stratified by the Coronary Artery Disease Reporting and Data System (CAD-RADS) classification in CCTA patients are summarized in Table 5 and Table 6. Patients without coronary artery stenosis (Group 1) were statistically significantly younger than patients with mild and moderate coronary artery stenosis (Groups 2 and 3) (*p* = 0.017 and *p* = 0.028, respectively). Patients with mild or minimal coronary artery stenosis (1–49%; Group 2) were significantly less likely to have arterial hypertension compared to patients without CAD (Group 1) and with moderate-to-severe coronary artery stenosis (50–100%; Groups 3 and 4) (*p* = 0.004).

Patients with moderate coronary artery stenosis (Group 3) were more likely to have dyslipidemia than others (*p* = 0.013). In addition, patients with the most severe CAD (Group 4) were more likely to have a family history of cardiovascular disease than patients without CAD (*p* = 0.045) (Table 5).

Use of angiotensin-converting enzyme (ACE) inhibitors or angiotensin II receptor blockers (ARBs) and beta-blockers was more common in patients with moderate or severe coronary artery stenosis (Groups 3 and 4) compared with those with mild or minimal stenosis or no stenosis (*p* = 0.041 and *p* = 0.020, respectively). Patients with mild or minimal stenosis (Group 2) used calcium channel blockers significantly less frequently than patients with severe stenosis (Group 4) (*p* = 0.031). No other differences in medication use were observed between groups based on the CAD-RADS score (Table 6).

### 2.2. Lipid Profiles of the Study Groups

Our study showed that, as expected, low-density lipoprotein cholesterol (LDL-C) and non-high-density lipoprotein cholesterol (non-HDL-C) levels were significantly higher in patients with CAD (*p* = 0.027 and *p* = 0.038, respectively), whereas other lipid parameters did not differ significantly (Table 7).

Based on CAD severity assessed using the Gensini score, a significant difference in oxLDL was observed, while no significant differences were found in the other lipid profile markers (Table 8). Specifically, patients with moderate and severe CAD (Groups 2 and 3) had significantly higher oxLDL levels compared to patients without CAD (Group 4). Furthermore, patients with moderate CAD (Group 2) exhibited significantly higher oxLDL levels than those with less severe CAD (Group 1) (Table 8).

Similarly, lipid markers differed among patients grouped by the CAD-RADS classification. As shown in Table 9, LDL-C and non-HDL-C levels were significantly higher in patients with moderate coronary stenosis (Group 3) than in those without coronary stenosis (Group 1). In contrast, oxLDL levels were similar across all CAD severity groups defined by CAD-RADS scores.

When patients were stratified by the presence of AH, median oxLDL levels were significantly higher in those with AH compared with those without AH (82.28 [33.33–257.56] vs. 67.98 [29.01–117.57]; *p* = 0.003, Mann–Whitney U test). OxLDL levels did not differ significantly according to gender or the presence of dyslipidemia.

Circulating oxLDL concentrations were also compared according to statin and ACE inhibitor/ARB use. OxLDL levels did not differ significantly between patients taking statins and those not taking statins. However, patients receiving ACE inhibitors or ARBs had significantly higher median oxLDL concentrations compared with those not taking these medications (83.74 [33.33–257.56] vs. 68.48 [29.01–121.43]; *p* = 0.002, Mann–Whitney U test).

### 2.3. The Differences in Vimentin and Endocan Levels in the Blood in the Patients, Grouped According to the Presence and Severity of CAD

Vimentin levels were significantly elevated in patients with CAD (*p* = 0.012). Conversely, endocan levels did not differ significantly between patients with and without CAD (Table 10).

Endocan and vimentin levels did not differ significantly among CAD patients stratified by Gensini score (Table 11). Nonetheless, a non-significant trend toward elevated vimentin levels was observed with increasing CAD severity.

A similar trend was observed among patients stratified by CAD-RADS score: although endocan and vimentin levels did not differ significantly, there was a tendency for vimentin levels to increase with greater CAD severity (Table 12).

Vimentin levels were also compared according to statin and ACE inhibitor/ARB use. Vimentin concentrations did not differ significantly between patients taking statins and those not taking them. However, patients using ACE inhibitors or ARBs had significantly higher median vimentin levels compared with non-users (224 [7.81–2081] vs. 150.42 [38.60–1485]; *p* = 0.049, Mann–Whitney U test).

When stratified by the presence of AH, patients with AH had higher median vimentin levels than those without AH (196.47 [7.81–2081] vs. 125.39 [38.60–656]; *p* = 0.046, Mann–Whitney U test). There were no significant differences in vimentin levels based on gender or the presence of dyslipidemia.

### 2.4. Differences in Serum Levels of Extracellular Matrix Glycoproteins (TSP-1 and TSP-2) Between Patients According to the Presence and Severity of CAD

TSP-1 and TSP-2 levels did not differ significantly according to CAD and its severity as assessed by Gensini and CAD-RADS scores (Table 13, Table 14 and Table 15).

### 2.5. Correlations

#### 2.5.1. Correlations Between Serum Lipid Markers and Selected Clinical Characteristics, Markers of Endothelial Damage, and Extracellular Matrix Glycoproteins

As shown in Table 16, significant positive correlations were observed between oxLDL levels and systolic blood pressure, as well as significant inverse correlations with ABI in both the left and right limbs. In addition, oxLDL showed statistically significant but very weak positive correlations with other lipid markers (total cholesterol, LDL-C, non-HDL-C) and with other investigated variables, including high-sensitivity C-reactive protein (hs-CRP), carotid IMT, and arterial stiffness, TSP-2 levels, and inverse correlation with endocan levels.

**Figure 1:**
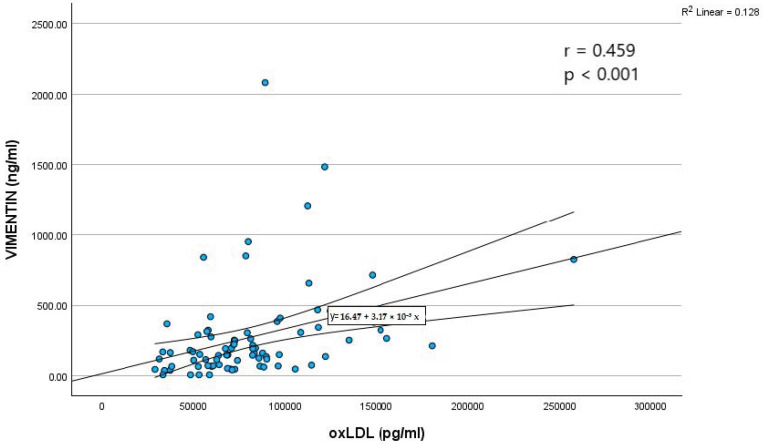
Spearman’s correlation between OxLDL and vimentin levels.

A moderate positive correlation between oxLDL and vimentin levels (r = 0.459, *p* < 0.001) was found (Figure 1).

Moderate positive correlation was found between TSP-1 and TSP-2 levels (r = 0.541, *p* < 0.001).

#### 2.5.2. Correlation Between Serum Lipids, Markers of Endothelial Damage, and Atherosclerosis Severity

Significant positive correlations were found between serum oxLDL levels and various indicators of atherosclerosis severity (Table 17).

Similarly, circulating vimentin levels showed significant correlations with all measured indices of atherosclerosis severity (Table 18). Together, these findings indicate that higher oxLDL and vimentin concentrations are associated with greater anatomical and clinical atherosclerotic burden as assessed by both invasive and non-invasive modalities.

## 3. Discussion

The novelty of our study lies in the comprehensive evaluation of lipid markers (Lp(a), ApoB, and oxLDL), endothelial injury markers (endocan, vimentin), and ECM glycoproteins (TSP-1 and TSP-2) in a carefully selected cohort of patients with SAP and without additional comorbidities. This integrative approach provides insight into the interplay between lipoprotein metabolism, endothelial dysfunction, and coronary atherosclerosis severity. Such findings may aid the future development of diagnostic biomarker panels for earlier detection of subclinical atherosclerotic changes that remain undetectable on conventional imaging.

### 3.1. Differences in OxLDL Levels Between CAD Groups

Our study demonstrated that, among lipid markers, oxLDL showed the strongest statistical association with increasing severity of CAD. Patients classified with moderate or severe CAD (Gensini Groups 2 and 3) exhibited significantly higher circulating oxLDL concentrations compared with patients with mild stenosis or angiographically confirmed absence of CAD (Table 8). These results are consistent with previous studies, such as Xu et al., who also demonstrated a positive association between the oxLDL/LDL-C ratio and CAD severity (odds ratio 1.83, 95% CI 1.21–2.77, *p* < 0.01) [27]. Unlike our cohort, their study population included 152 patients with diabetes mellitus, but similar to our study, they also used the Gensini scoring system to classify CAD severity. In contrast, our cohort excluded major comorbidities, allowing for clearer interpretation of oxLDL–CAD relationships.

Additional studies in acute coronary syndrome (ACS) cohorts have likewise demonstrated that greater atherosclerotic coronary burden is associated with significantly elevated oxLDL concentrations [28,29]. While these studies focused on acute conditions, our findings extend the evidence by demonstrating strong oxLDL and CAD associations in a stable, comorbidity-free population. Gruzdeva et al. reported that oxLDL levels were significantly higher in patients with multivessel CAD compared to patients with single-vessel disease, highlighting its potential as a marker of disease severity in patients with ACS [28]. Their study sample was quite large, comprising 400 patients with ST-elevation ACS. Similarly, Ehara et al. studied circulating oxLDL levels in 135 patients with ACS, unstable angina pectoris, or stable angina pectoris, and healthy control subjects. Their study showed that oxLDL levels in patients with ACS were almost four times higher than in the control group, indicating a strong association between oxidized LDL and ACS and their severity [29]. The design of these studies highlights the role of oxLDL in plaque instability and more severe coronary lesions in ACS.

### 3.2. Correlations Between OxLDL and Gensini Score Groups

We identified significant positive correlations between oxLDL levels and multiple angiographic markers of CAD severity, including the Gensini score, the number of atherosclerotic coronary arteries and involved segments, the presence of significant ramus circumflexus (RCx) stenosis, and the need for PCI (Table 17). These associations are consistent with current concepts linking LDL oxidative modification to a more atherogenic lipid milieu and increased oxidative stress. Poznyak et al. proposed that oxidative modification of LDL within the vascular endothelium represents an early event in atherosclerotic plaque development, driven by an imbalance between ROS and endogenous antioxidant defenses. OxLDL is known to participate in processes such as foam cell formation and immune activation, which contribute to plaque progression and vascular inflammation [30]. Although oxidative modification of LDL is integral to atherogenesis [3], the present findings should be interpreted strictly as correlations rather than evidence of causality. Importantly, these relationships were observed in clinically stable patients without comorbidities, thereby minimizing potential confounding factors.

### 3.3. OxLDL and Vascular Function Indicators

Patients with higher oxLDL levels displayed more adverse vascular phenotypes, including higher systolic blood pressure and lower ABI values. These observations support the potential utility of oxLDL as a marker that correlates with subclinical or more advanced vascular alterations. OxLDL showed a significant positive association with systolic blood pressure in our cohort. Similar correlations were reported by Cicero et al., who found that oxLDL was associated with systolic blood pressure, ApoB, and serum uric acid in a cohort of 417 patients with and without chronic kidney disease [31]. By excluding renal impairment and other major comorbidities, our study confirms this correlation in a more homogeneous population and demonstrates that patients with arterial hypertension tend to have higher oxLDL levels. These findings should, however, be interpreted as associations rather than evidence of a causal relationship.

OxLDL was inversely associated with ABI, suggesting a possible link with early peripheral arterial changes. This pattern is consistent with previous studies by Holvoet et al., who observed that higher oxLDL levels were associated with subclinical PAD and atherosclerotic progression in an international cohort [32]. Our results extend these associations to a non-diabetic, comorbidity-free CAD population. Overall, the demonstrated association confirms the potential value of oxLDL as an integrated marker reflecting vascular changes. It is important to note that these associations do not imply a causal relationship and should be interpreted in the context of observational data.

Recent evidence suggests that arterial stiffness and carotid IMT may improve early detection and risk stratification of atherosclerosis [33]. In our study, only oxLDL demonstrated a very weak positive correlation with carotid IMT, indicating a possible, but limited, association with subclinical vascular changes. These findings align with those of Metso et al., who similarly reported an association between oxLDL levels and carotid IMT in subclinical carotid atherosclerosis [34]. However, this relationship must be interpreted with caution due to the small sample size and the weak correlation coefficient. Larger, well-powered studies are needed to further evaluate and validate this association.

### 3.4. Correlations Between Vimentin and CAD Severity

Circulating vimentin concentrations in our study were significantly associated with multiple indicators of CAD severity, including the Gensini score, segment involvement score (SIS), segment stenosis score (SSS), CAD-RADS, the number of diseased arteries, and the requirement for PCI. These findings are consistent with earlier work showing the relevance of vimentin in cardiovascular pathology. Kondo et al. analyzed 26 formalin-fixed myocardial samples from patients with CAD, and demonstrated that vimentin highlights endothelial–mesenchymal transition and myocardial remodeling, underscoring its value as a tissue-level marker of CAD-related structural change [21]. Although focused on histology, their work supports the broader involvement of vimentin in vascular injury.

Gong et al. provided complementary evidence through a clinical cohort of 288 CAD and 195 non-CAD patients, reporting higher circulating vimentin levels in CAD and a positive association with the extent of coronary involvement. Their in vitro experiments showed that recombinant vimentin enhances adhesion molecule and cytokine expression in endothelial cells and macrophages, and their ApoE^−/−^ mouse studies demonstrated that vimentin contributes to inflammatory responses relevant to atherosclerosis [35]. Our findings are consistent with the clinical component of these studies, indicating that higher vimentin levels correspond to greater CAD burden across several independent severity metrics. Taken together, these findings suggest that increased circulating vimentin in the presence of elevated oxLDL may reflect shared cellular stress and remodeling responses within the vascular wall, rather than a direct causal effect.

Mechanistic and histological studies offer an important context for these associations. Shakhov et al. reported increased vimentin expression in atherosclerotic lesions, highlighting its involvement in cytoskeletal reorganization and intercellular communication within regions of active remodeling [36]. Vimentin has been implicated in endothelial–mesenchymal transition, cytoskeletal remodeling, and interactions with the ECM [37]. Experimental models further show that vimentin expression responds to lipid and inflammatory stress. He et al. demonstrated that miR-144 knockout mice exhibit increased secretory vimentin, altered cholesterol metabolism via ABCA1, and enhanced aortic vimentin accumulation, linking vimentin to both lipid handling and vascular remodeling processes [37]. Although these mechanistic findings come from animal models, they illustrate how vimentin responds to metabolic and inflammatory cues relevant to atherosclerosis.

Our findings are consistent with broader evidence linking vimentin to vascular inflammation and remodeling. Overall, these observations suggest that elevated circulating vimentin may reflect parallel oxidative, inflammatory, and structural remodeling processes within the vascular wall, rather than indicating a direct causal role in disease progression. In our cohort, however, vimentin did not show significant associations with the vascular function indicators assessed (carotid IMT, ABI, and arterial stiffness) or with the ECM glycoproteins TSP-1 and TSP-2. This absence of correlation may be related to the relatively small sample size, the inclusion of patients with stable angina only—representing earlier or less active stages of atherosclerotic disease—and the inherent variability of circulating biomarker measurements.

The inclusion of both ICA and CCTA reflects contemporary real-world clinical practice in the diagnostic evaluation of CAD presence and severity [38,39]. The use of different imaging modalities may have introduced heterogeneity in the observed associations between circulating biomarkers and CAD severity and should therefore be considered when interpreting the results. Increasing evidence suggests that integration of circulating biomarkers with CCTA-derived assessments may provide complementary molecular and anatomical information for CAD detection and risk stratification in patients with suspected disease, as well as in population-based cohorts and in acute or chronic coronary syndromes [38]. Larger, prospective studies employing standardized imaging protocols and analytical frameworks are required to clarify the biological and clinical relevance of combined biomarker-based and imaging-based diagnostic approaches, including CCTA and ICA.

### 3.5. Discussion of Extracellular Matrix Glycoproteins (TSP-1 and TSP-2) in Relation to CAD Severity

#### 3.5.1. Differences in TSP-1 and TSP-2 Levels Between the Groups

We evaluated circulating concentrations of the ECM glycoproteins TSP-1 and TSP-2, whose roles in atherosclerosis remain incompletely understood and, in some cases, controversial. TSP-1 has been implicated in endothelial cell apoptosis, modulation of vasa vasorum remodeling, platelet aggregation, and attenuation of nitric oxide–mediated vasodilation [25]. In contrast, the function of TSP-2 in atherosclerosis is less clearly defined, and available data suggest diverse context-dependent effects [26].

In our study, circulating TSP-1 and TSP-2 levels did not differ significantly between patients with and without CAD, nor across groups stratified by CAD severity (CAD-RADS or Gensini score; Table 13, Table 14 and Table 15). These findings are consistent with those of Choi et al., who reported higher TSP-1 concentrations in CAD patients with diabetes mellitus (*n* = 103) compared with individuals without CAD or diabetes (*n* = 108) (564.5 ± 113.5 ng/mL vs. 515.5 ± 127.4 ng/mL; *p* < 0.001), but found no significant differences when patients were classified solely by CAD status and no correlation with Gensini scores [23]. In our metabolically healthier cohort, which excluded diabetes mellitus and other major comorbidities, we likewise observed no significant relationship between TSP-1 levels and CAD. Taken together, these observations suggest that circulating TSP-1 may be more strongly influenced by metabolic comorbidities—particularly diabetes mellitus—than by the presence or severity of CAD alone.

#### 3.5.2. Correlations Between TSP-1 and TSP-2 and Other Markers

Although no group-level differences were observed, our study identified a significant moderate positive correlation between circulating TSP-1 and TSP-2 levels. Previous genetic studies by Boekholdt et al. demonstrated that certain thrombospondin gene polymorphisms were associated with a reduced risk of early myocardial infarction, possibly via regulation of matrix metalloproteinase-2 and enhanced ECM stability [26]. However, in contrast to these genetic associations, our study did not detect differences in circulating TSP-1 or TSP-2 based on CAD status or severity.

#### 3.5.3. TSP-1, TSP-2, and CAD Severity: Comparison with Mechanistic and Tissue Studies

Recent mechanobiological data provide additional context. Zhao et al. reported that TSP-1 acts as a mechanosensitive mediator: under physiological mechanical stress, it is secreted at low levels, whereas pathological hemodynamic forces markedly upregulate TSP-1 expression, which in turn modulates endothelial adhesion, migration, proliferation, and collagen deposition—processes contributing to vascular dysfunction and atherosclerotic remodeling [40]. TSP-2, conversely, appears to support extracellular matrix stability and exert anti-remodeling effects, although its role remains incompletely characterized [41].

Tissue-level evidence from Sonmez et al. showed that TSP-2 expression was significantly lower in atherosclerotic aortic tissue than in non-atherosclerotic internal mammary artery samples, suggesting that reduced TSP-2 expression may accompany extracellular matrix destabilization [41]. These tissue-based findings differ from our results, as we did not observe significant differences in circulating TSP-2 between patients with and without CAD. This discrepancy may reflect differences between circulating biomarkers and local tissue expression, as well as variations in disease stage.

Taken together, our findings suggest that although TSP-1 and TSP-2 participate in biological processes related to extracellular matrix remodeling and vascular injury, their circulating concentrations in stable CAD appear to be more strongly influenced by concomitant conditions and acute inflammatory settings than by the severity of coronary atherosclerosis itself. The absence of significant associations in our cohort likely reflects several factors, including the stable-angina population studied, the relatively small sample size, and the known variability of circulating ECM glycoproteins.

### 3.6. Discussion on Statins and ACE Inhibitors’ Effect on Lipid and Endothelial Markers

Although statins are well known for their potent LDL-C-lowering effect, their influence on Lp(a) remains unclear. Data from various studies are conflicting: some studies show that statin use increases Lp(a), others show a decrease, and most studies show no significant changes [42,43]. A systematic review and meta-analysis by De Boer et al. conclude that statins do not reduce Lp(a) levels to a clinically significant extent, while a large retrospective cohort study by Feng et al. involving 42,166 Chinese patients showed that statin use is associated with a higher risk of Lp(a) elevation, and this result has been replicated in several balanced cohort studies [42,43]. These observations appear to be independent of statin intensity or lipophilicity and likely reflect pleiotropic effects on hepatic lipoprotein metabolism, inflammation, and oxidative stress rather than a direct Lp(a)-lowering mechanism [44,45]. The reduction in hs-CRP, which is known to be pathophysiologically related to oxLDL, together with the reduction in the amount of LDL particles available for oxidation, may contribute to the reduction in oxLDL formation [46]. In summary, although the effect of statins on Lp(a) remains unclear and requires extensive real-world studies, the evidence clearly supports a positive effect on oxLDL, adding to the cardioprotective profile of statin therapy.

In our study, oxLDL levels did not differ significantly between statin users and non-users. This result contradicts larger meta-analyses, which show a clear reduction in oxLDL with statin use, and may be explained by the limited sample size of our study, which reduces the ability to detect such differences. Therefore, larger real-world studies are needed to better define the relationship between statin use and oxLDL levels.

Previous studies suggest that ACE inhibitors may improve endothelial function by acting on the balance between angiotensin II and nitric oxide, the main mechanism regulating vascular tone, hemostasis, and vascular structure [47,48]. In our study, the marker of endothelial dysfunction, vimentin, was higher in patients with arterial hypertension, including those treated with ACE inhibitors or ARBs, suggesting that endothelial damage may persist despite antihypertensive treatment. Vimentin levels did not differ according to gender or dyslipidemia status. OxLDL levels were also higher in patients with AH, confirming the influence of oxidative stress on its pathogenesis. However, we did not evaluate drug dosage or duration of use, which limits the interpretation of these associations. Future large-scale studies with detailed information on treatment duration and dosages are needed to clarify these results.

### 3.7. Correlation Between oxLDL and Endothelial Damage Markers

OxLDL is known to engage endothelial stress-related pathways, including those mediated by the lectin-like oxidized LDL receptor-1, which has been implicated in reduced nitric oxide bioavailability and endothelial dysfunction [3,27,29,30]. In the present study, oxLDL levels were correlated with circulating markers of endothelial injury; however, these observations should be interpreted strictly as associations rather than evidence of direct mechanistic effects.

#### 3.7.1. Correlations Between oxLDL and Vimentin

We observed a moderate positive association between oxLDL and circulating vimentin levels. Recent experimental evidence provides a mechanistic context for this relationship. Martínez-Cenalmor et al. demonstrated that oxidative stress induces rapid and reversible remodeling of vimentin filaments into biomolecular condensates, highlighting vimentin’s sensitivity to redox imbalance [49]. Because oxLDL is known to promote oxidative and inflammatory activation in endothelial cells and macrophages, the concurrent elevation of oxLDL and vimentin in our cohort may reflect parallel cellular stress responses within the vascular milieu. Additional studies describing oxLDL-induced extracellular vimentin release from macrophages and oxidative stress–dependent vimentin reorganization further support this interpretation [20,50]. Taken together, these findings suggest that the oxLDL–vimentin association observed in our study could reflect shared involvement in oxidative and inflammatory pathways relevant to atherosclerosis.

Our results are also in agreement with the findings of Kim et al., who reported higher circulating vimentin levels in CAD patients compared with controls and identified a strong positive correlation between oxLDL and vimentin concentrations (r = 0.6538) in a cohort that included individuals with diabetes mellitus [20]. In our comorbidity-free population, we similarly observed a significant positive association between oxLDL and vimentin and a nearly 2.6-fold increase in circulating vimentin levels among CAD patients. Although Kim et al. demonstrated that oxLDL can stimulate vimentin secretion through CD36-mediated signaling in experimental models, our observational data do not allow causal inference. Instead, the consistency between experimental and clinical findings supports the interpretation that elevated oxLDL and vimentin may co-occur as part of broader oxidative and inflammatory responses characteristic of atherosclerotic disease.

Recent evidence further supports the biological relevance of vimentin in vascular pathology. Vimentin coordinates actin stress fibers and podosome dynamics in macrophages, contributing to ECM–cell interactions and matrix remodeling, and concentrated vimentin networks respond to areas of active ECM degradation [50]. In atherosclerotic lesions, extracellular vimentin is enriched in regions with abundant activated inflammatory cells, and circulating vimentin levels are higher in individuals with CAD than in healthy controls [51]. In addition, extracellular and membrane-associated vimentin has been shown to participate in cell–cell communication and tissue-remodeling processes, providing further context for its elevation alongside oxLDL. Evidence from cancer biology also demonstrates that vimentin is highly responsive to cellular stress and regulates cytoskeletal remodeling, migratory behavior, and inflammatory activation—features that reflect a broader principle applicable across pathologies: vimentin expression increases in settings of oxidative stress and structural remodeling [52]. Within the vascular wall, such properties may reflect endothelial stress or macrophage activation. Taken together, these insights support the interpretation that elevated vimentin and oxLDL in our cohort likely represent parallel oxidative and inflammatory responses rather than a direct causal interaction.

OxLDL is a key driver of oxidative stress-mediated vascular injury, promoting endothelial activation through the induction of adhesion molecules such as intercellular adhesion molecule-1 (ICAM-1), vascular cell adhesion molecule-1 (VCAM-1) and E-selectin, and facilitating macrophage uptake via macrophage scavenger receptors (CD3, MSR-1), leading to foam cell formation and plaque destabilization [34]. Vimentin, increasingly recognized as a marker of endothelial activation and cytoskeletal stress, may act in parallel with oxLDL-related oxidative pathways, supporting leukocyte adhesion and amplifying local inflammation [20,22]. In this context, the positive association between oxLDL and vimentin observed in our study suggests coordinated involvement in vascular injury processes.

Compared to established systemic markers such as hs-CRP, which primarily reflect general inflammatory burden, oxLDL and vimentin may offer greater specificity for oxidative endothelial stress and plaque-level activation [53]. The weak correlation between oxLDL and hs-CRP in our cohort, consistent with previous reports, reinforces that systemic inflammation does not necessarily mirror lipid oxidation within the arterial wall. Similarly, VCAM-1 and P-selectin serve as circulating surrogates of endothelial adhesion, yet may decline to capture oxidative lipoprotein modification [54]. Thus, oxLDL and vimentin may complement—not replace—conventional biomarkers by highlighting the oxidative and structural aspects of vascular injury that systemic markers such as hs-CRP reflect only indirectly.

#### 3.7.2. Discussion on CAD and Endocan

In our study, endocan levels did not differ significantly between patients with and without CAD or across groups stratified by atherosclerosis severity. Endocan has gained attention as an endothelial dysfunction marker due to its involvement in vascular smooth muscle cell proliferation and migration—processes relevant to neointimal formation in atherosclerosis [3,16]. Kose et al. reported significantly higher serum endocan concentrations in patients with ACS, particularly among those with diabetes mellitus, and observed correlations with the number of affected coronary arteries, although no associations were found with Gensini or SYNTAX scores [16]. These findings are supported by more recent analyses. For instance, a recent meta-analysis demonstrated that circulating endocan levels were significantly higher in patients with ACS compared with controls, indicating its potential relevance as a biomarker of endothelial dysfunction and myocardial injury during acute events. Similarly, several ACS cohort studies have shown that higher endocan levels at admission are associated with an increased risk of major adverse cardiac events (MACEs) and with higher clinical risk scores such as thrombolysis in myocardial infarction [55,56].

In contrast, our cohort consisted exclusively of patients with SAP, which may partly explain the absence of significant endocan and CAD associations in our results. Additional factors, including the relatively small sample size and the known variability of circulating endocan concentrations, may also have limited the detection of subtle differences. Chen et al. emphasized that the diagnostic value of endocan is limited when assessed in isolation due to its structural complexity and variable expression, recommending that it be interpreted alongside other established endothelial or inflammatory markers [57]. Taken together, these observations suggest that endocan may be more informative in acute coronary syndromes or in earlier, more dynamic stages of endothelial dysfunction, whereas its utility in stable CAD remains uncertain. Importantly, the associations observed in our study are correlational and should not be interpreted as causal, and further research is warranted to clarify the clinical relevance of endocan across different stages of atherosclerosis.

## 4. Materials and Methods

### 4.1. Study Population

This cross-sectional pilot study included 93 consecutive patients with SAP evaluated at the Cardiology Clinic of the Lithuanian University of Health Sciences Hospital Kaunas Clinics between October 2023 and December 2024.

Exclusion criteria comprised age < 18 years; history of ACS, prior coronary revascularization, PAD, heart failure or left ventricular ejection fraction < 50%; significant valvular disease; acute/chronic infections, inflammatory or autoimmune disease; recent trauma or surgery; glucocorticoid therapy; malignancy, hematologic, endocrine, hepatic or renal disease (estimated glomerular filtration rate < 60 mL/min).

Baseline demographics, cardiovascular risk factors, comorbidities, and medication use were obtained from electronic health records. Smoking status and family history of premature cardiovascular disease were recorded.

The study was approved by the Regional Bioethics Committee of the Lithuanian University of Health Sciences (No. BE-2-132) and conducted in accordance with the Declaration of Helsinki and the US Code of Federal Regulations, Title 45, Part 46, “Protection of Human Subjects” (revised 15 January 2009, entered into force on 14 July 2009).

### 4.2. Laboratory Measurements

Fasting venous blood serum samples were collected prior to diagnostic testing. Standard biochemical analyses (lipid profile, ApoB, Lp(a), hs-CRP, uric acid) were performed using the AU680 automated clinical chemistry analyzer (Beckman Coulter, Brea, CA, USA). Complete blood counts were performed using the UniCel DxH 800 automated hematology analyzer (Beckman Coulter, Brea, CA, USA).

Serum aliquots were stored at −80 °C for the determination of oxLDL, endocan, vimentin, TSP-1, and TSP-2. These biomarkers were measured using commercial ELISA kits according to the manufacturers’ protocols (using the immunoenzymatic method with the EUROIMMUN Analyzer I (EUROIMMUN Medizinische Labordiagnostika AG, Lübeck, Germany) and commercial ELISA kits Human OxLDL (Oxidized Low-Density Lipoprotein) ELISA Kit, Human VIM (Vimentin) ELISA kit and Human ESM1 (endothelial cell specific molecule 1) ELISA kit (catalog# E-EL-H6021, catalog# E-EL-H1094 and catalog# E-EL-H1557, respectively, from Elabscience Biotechnology Inc., Houston, TX, USA); Human THBS2 (thrombospondin-2) ELISA kit and Human TSP-1 (thrombospondin-1) ELISA kit (catalog# ELK1335 and catalog# ELK10311, respectively, from ELK Biotechnology, Wuhan, China).

### 4.3. Instrumental Examination

Cardiac structure and function were assessed by transthoracic echocardiography. Coronary atherosclerosis severity was assessed by ICA and/or CCTA. CCTA was used in patients with low–low–intermediate pre-test probability, while ICA was performed in those with high clinical likelihood, persistent symptoms, or when CCTA suggested obstructive disease.

Carotid ultrasound was performed to measure IMT and detect plaques; IMT > 0.9 mm or plaque presence was considered abnormal. Arterial stiffness was evaluated using CAVI and PWV, with CAVI > 9.0 and PWV > 10 m/s deemed abnormal [58]. ABI < 0.9 or >1.40 was considered pathological [59].

CAD severity on CCTA was graded using the CAD-RADS classification: CAD-RADS 0: no plaque; CAD-RADS 1–2: minimal–mild stenosis (1–49%); CAD-RADS 3: moderate stenosis (50–69%); CAD-RADS 4–5: severe stenosis (≥70%) or occlusion [60].

Semi-quantitative indices, including the SIS and SSS, were calculated to quantify overall plaque burden. SIS was defined as the total number of coronary segments containing any detectable plaque on CCTA. SSS was calculated by summing the stenosis grades of each coronary segment, based on luminal narrowing severity: 0 = no plaque/stenosis, 1 = mild stenosis < 50%, 2 = moderate stenosis 50–69%, and 3 = severe stenosis ≥ 70% [61].

Invasive angiograms were evaluated using the Gensini scoring system, which grades stenosis severity and weights each segment according to anatomical importance [62]. The total score reflects the overall CAD burden.

First, patients were divided into two groups based on ICA and/or CCTA findings: Non-CAD group: no coronary stenosis (*n* = 22); CAD group: any degree of stenosis (*n* = 71; 76.3%).

Second, patients were categorized by severity according to the imaging modality performed:

(1) ICA (Gensini score): No stenosis (Gensini score 0): *n* = 7; Mild (Gensini score 0–10): *n* = 10; Moderate (Gensini score 11–30): *n* = 9; Severe (Gensini score ≥ 31): *n* = 9;
(2) CCTA (CAD-RADS classification): CAD-RADS 0 (no stenosis): *n* = 18; CAD-RADS 1–2 (minimal–mild): *n* = 35; CAD-RADS 3 (moderate): *n* = 12; CAD-RADS 4–5 (severe): *n* = 13.

### 4.4. Statistical Analysis

Statistical analyses were performed using IBM SPSS Statistics 30.0. Normality was assessed using the Kolmogorov–Smirnov and Shapiro–Wilk tests. Continuous variables are presented as means and standard deviations or medians with ranges of minimum and maximum values as appropriate; categorical variables are expressed as counts and percentages.

Group comparisons employed Student’s *t*-test or Mann–Whitney U test for continuous variables and the chi-square test for categorical variables. Differences across multiple severity groups were assessed using the Kruskal–Wallis test. Spearman’s rank correlation coefficient was applied to examine associations among lipid biomarkers, markers of endothelial injury, and ECM, as well as oxidative stress markers and CAD severity. Statistical significance was defined as a two-tailed *p* value < 0.05.

## 5. Conclusions

This pilot, hypothesis-generating study provides an integrated evaluation of lipid-related biomarkers, endothelial injury markers, and ECM glycoproteins in a relatively homogeneous cohort of patients with SAP and without major comorbidities. Among the biomarkers examined, oxLDL demonstrated the strongest associations with CAD severity, correlating with Gensini scores, the extent of coronary involvement, systolic blood pressure, and ABI. Elevated vimentin levels were also consistently associated with a greater atherosclerotic burden, supporting its potential pathophysiological relevance as a marker of endothelial stress and vascular remodeling.

In contrast, endocan and ECM glycoproteins (TSP-1, TSP-2) showed no significant association with CAD presence or severity, suggesting that their circulating levels may be less sensitive to stable atherosclerotic changes or influenced by disease-modifying factors absent in this cohort. The observed relationship between oxLDL and vimentin highlights a potential link between oxidative stress, endothelial injury, and vascular remodeling that warrants further mechanistic investigation.

Given the exploratory nature of this single-center study and its limited sample size, these findings should be interpreted with caution. They are intended to generate hypotheses rather than establish clinical utility, and require confirmation in larger, prospective, multi-center cohorts with longitudinal follow-up and mechanistic characterization before any clinical implications can be drawn.

This study has several limitations. The sample size was relatively small and derived from a single center, which may limit generalizability. The cross-sectional design allows only correlational interpretation and precludes causal inference. Plaque composition and calcification were not assessed, as intravascular ultrasound, optical coherence tomography, calcium scoring, or detailed plaque characterization by CCTA were not performed. Information on medication dosage and treatment duration was unavailable, limiting the assessment of pharmacological effects on biomarker levels and introducing the possibility of residual confounding by medication use. Moreover, residual confounding by traditional cardiovascular risk factors cannot be excluded despite careful clinical characterization of the cohort. In addition, single-time-point biomarker measurements may not capture temporal variability, and the lack of assay standardization for oxLDL may affect comparability across studies. Finally, the absence of long-term follow-up precluded evaluation of clinical outcomes, such as MACEs or mortality.

## Figures and Tables

**Table 1:** Baseline characteristics of patient groups according to CAD presence.

	CAD Group (*n* = 71)	Non-CAD (*n* = 22)	*p* Value
Age, years	57.79 (10.61)	51 (14)	*0.039* *
Female gender	34 (47.9)	14 (63.6)	*0.229*
BMI, kg/m^2^	29.44 (5.18)	26.32 (4.88)	*0.028* *
Smoking	20 (28.2)	5 (22.7)	*0.785*
Obesity	30 (42.3)	5 (22.7)	*0.234*
Arterial hypertension	50 (70.4)	14 (63.6)	*0.602*
Dyslipidemia	66 (93.0)	14 (63.6)	*0.002*
Early family history of cardiovascular disease	15 (21.1)	0 (0)	*0.018*
Systolic BP, mmHg	143.09 (17.58)	134.14 (20.56)	*0.026* *
Diastolic BP, mmHg	88.11 (11.01)	84.43 (12.8)	*0.152* *
Heart rate, bpm	69.64 (9.96)	69.95 (7.71)	*0.529* *
Uric acid, μmol/L	325.93 (76.25)	310.91 (76.89)	*0.415* *
Abnormal IMT	20 (31.3)	5 (25.0)	*0.781*
Abnormal CAVI	23 (46.9)	5 (25.0)	*0.390*
Anormal PWV	1 (1.4)	0 (0)	*0.527*
Suspected PAD	10 (15.6)	0 (0)	*0.107*

* Student’s *t*-test, data are presented as mean (standard deviation). Chi-square test for homogeneity everywhere else, data are presented as *n* (%). BMI, body mass index; BP, blood pressure; bpm, beats per minute; abnormal IMT, intima–media thickness more than 0.9 mm; abnormal CAVI, measurement of the cardio-ankle vascular index higher than 9.0; abnormal PWV, pulse wave velocity higher than 10 m/s; suspected PAD, suspected peripheral arterial disease—ABI less than 0.9. The dyslipidemia group included patients with total cholesterol >5 mmol/L, LDL-C > 3 mmol/L, triglycerides > 1.7 mmol/L, HDL-C < 1.0 mmol/L for men or <1.2 mmol/L for women, as well as those receiving statin therapy regardless of achieved lipid values. Reference intervals for uric acid: men 208.3–428.4 μmol/L; women 154.7–357 μmol/L.

**Table 2:** Medication use in patient groups according to CAD presence.

	CAD Group (*n* = 71)	Non-CAD (*n* = 22)	*p* Value
Statin	26 (36.6)	6 (27.3)	*0.456*
ACE inhibitor/ARB	37 (52.1)	9 (40.9)	*0.465*
Beta-blocker	32 (45.1)	8 (36.4)	*0.623*
Calcium channel blocker	11 (15.5)	4 (18.2)	*0.748*

ACE, angiotensin-converting enzyme; ARB, angiotensin II receptor blocker. Chi-square test for homogeneity was used. Results are presented as *n* (%).

**Table 3:** Baseline characteristics of patients who underwent ICA, categorized according to Gensini score.

	Groups by Gensini Score			Group 4 (Patients Without CAD) (*n* = 7)	*p* Value
	Group 1 (*n* = 10)	Group 2 (*n* = 9)	Group 3 (*n* = 9)		
Age, years	61 (57–80)	71 (48–80)	64 (46–75)	61 (51–66)	*0.335* *
Female gender	6 (60.0)	6 (66.7)	3 (33.3)	3 (42.9)	*0.472*
BMI, kg/m^2^	28.83 (23.80–41.03)	27.83 (21.55–36.33)	28.08 (24.80–33.21)	28.72 (22.41–33.41)	*0.970* *
Smoking	2 (20.0)	2 (22.2)	6 (66.7)	1 (14.3)	*0.069*
Obesity	4 (40.0)	4 (44.4)	4 (44.4)	3 (42.9)	*0.935*
Arterial hypertension	9 (90.0) ^a^	9 (100.0) ^a^	7 (77.8)	3 (42.9) ^a^	*0.030*
Dyslipidemia	10 (100.0)	9 (100.0)	9 (100.0)	6 (85.7)	*0.249*
Early family history of cardiovascular disease	1 (10.0)	2 (22.2)	4 (44.4)	0 (0.0)	*0.124*
Systolic BP, mmHg	154.5 (120–176)	152 (125–170)	144 (122–161)	130 (100–164)	*0.597* *
Diastolic BP, mmHg	84 (70–100)	88 (68–108)	88 (64–102)	80 (70–110)	*0.990* *
Heart rate, bpm	66 (56–90)	68 (59–84)	68 (57–95)	63 (50–83)	*0.717* *
Uric acid, μmol/L	323 (189–375)	283 (202–340)	374 (276–453)	338 (264–491)	*0.084* *
Abnormal IMT	3 (30.0)	5 (71.4)	3 (42.9)	0 (0.0)	*0.060*
Abnormal CAVI	5 (62.5)	5 (55.6)	4 (50.0)	2 (28.6)	*0.878*
Anormal PWV	1 (10.0)	0 (0.0)	0 (0.0)	0 (0.0)	*0.413*
Suspected PAD	0 (0) ^b^	6 (66.7) ^b^	2 (22.0) ^b^	0 (0.0) ^b^	*0.003*

* Kruskal–Wallis test, data are presented as medians (minimum–maximum). Chi-square test for homogeneity everywhere else, data are presented as *n* (%). Superscript letters (a,b) indicate statistically significant differences between groups according to the Z-test with Bonferroni correction (*p* < 0.05). BMI, body mass index; BP, blood pressure; bpm, beats per minute; abnormal IMT, intima–media thickness more than 0.9 mm; abnormal CAVI, measurement of the cardio-ankle vascular index higher than 9.0; abnormal PWV, pulse wave velocity higher than 10 m/s; suspected PAD, suspected peripheral arterial disease—ABI less than 0.9. The dyslipidemia group included patients with total cholesterol > 5 mmol/L, LDL-C > 3 mmol/L, triglycerides > 1.7 mmol/L, HDL-C < 1.0 mmol/L for men or <1.2 mmol/L for women, as well as those receiving statin therapy regardless of achieved lipid values. Reference intervals for uric acid: men 208.3–428.4 μmol/L; women 154.7–357 μmol/L.

**Table 4:** Medication use in patients who underwent ICA, grouped by Gensini score.

	Group 4 (Patients Without CAD) (*n* = 7)	Groups by Gensini Score			*p* Value
		Group 1 (*n* = 10)	Group 2 (*n* = 9)	Group 3 (*n* = 9)	
Statin	2 (28.6)	2 (20.0)	4 (44.4)	6 (66.7)	*0.188*
ACE inhibitor/ARB	3 (42.9)	5 (50.0)	9 (100.0)	5 (55.6)	*0.059*
Beta-blocker	4 (57.1)	6 (60.0)	7 (77.8)	6 (66.7)	*0.811*
Calcium channel blocker	0 (0.0)	2 (20.0)	3 (33.3)	1 (11.1)	*0.334*

ACE, angiotensin-converting enzyme; ARB, angiotensin II receptor blocker. Chi-square test for homogeneity was used. Results are presented as *n* (%).

**Table 5:** Baseline characteristics of patients who underwent CCTA, categorized according to CAD-RADS score.

	Groups by CAD-RADS Score				*p* Value
	Group 1 (*n* = 18)	Group 2 (*n* = 35)	Group 3 (*n* = 12)	Group 4 (*n* = 13)	
Age, years	52 (18–79) ^a,b^	56 (30–61) ^b^	58 (47–80) ^a^	62 (44–77)	*0.004* * 0.017 ^b^ *0.028* ^a^
Female gender, *n* (%)	12 (66.7)	14 (40.0)	8 (66.7)	6 (46.2)	*0.190* *
BMI, kg/m^2^	26.8 (17.9–38.1)	29.2 (21.7–46.9)	29.8 (23.8–40.1)	29.3 (20.0–35.4)	*0.136* *
Smoking, *n* (%)	4 (22.2)	9 (25.7)	2 (16.7)	6 (46.2)	*0.346*
Obesity, *n* (%)	4 (22.2)	13 (37.1)	6 (50.0)	6 (46.2)	*0.573*
Arterial hypertension, *n* (%)	13 (72.2) ^e^	17 (48.6) ^c^	12 (100.0) ^c,d,e^	11 (84.6) ^d^	*0.004*
Dyslipidemia, *n* (%)	11 (61.1) ^f^	31 (88.6) ^f^	12 (100.0) ^f^	12 (92.3) ^f^	*0.013*
Early family history of cardiovascular disease, *n* (%)	0 (0.0) ^g^	6 (17.1)	2 (16.7)	5 (38.5) ^g^	*0.045*
Systolic BP, mmHg	134 (110–180)	138 (104–185)	145 (126–176)	150 (125–170)	*0.114* *
Diastolic BP, mmHg	86 (60–106)	88 (78–120)	89 (70–113)	88 (64–100)	*0.408* *
Heart rate, bpm	70 (56–79)	68 (50–100)	66 (59–86)	68 (56–86)	*0.900* *
Uric acid, μmol/L	310 (154–419)	313 (154–482)	319 (189–485)	374 (231–453)	*0.527* *
Abnormal IMT, *n* (%)	5 (29.4)	6 (18.2)	5 (45.5)	3 (33.3)	*0.331*
Abnormal CAVI, *n* (%)	4 (33.3)	7 (33.3)	3 (42.9)	4 (40.0)	*0.957*
Anormal PWV, *n* (%)	0 (0.0)	0 (0.0)	1 (8.3)	0 (0.0)	*0.357*
Suspected PAD, *n* (%)	0 (0.0)	1 (3.1)	2 (20.0)	1 (9.1)	*0.150*

* Kruskal–Wallis test, data are presented as medians (minimum–maximum). Superscript letters (a,b,c,d,e,f,g) indicate statistically significant differences between groups based on pairwise post hoc comparisons with Bonferroni correction following the Kruskal–Wallis test (*p* < 0.05). Chi-square test for homogeneity everywhere else, data are presented as *n* (%). Superscript letters (c,d,e,f,g) indicate statistically significant differences between groups according to the Z-test with Bonferroni correction (*p* < 0.05). BMI, body mass index; BP, blood pressure; bpm, beats per minute; abnormal IMT, intima–media thickness more than 0.9 mm; abnormal CAVI, measurement of the cardio-ankle vascular index higher than 9.0; abnormal PWV, pulse wave velocity higher than 10 m/s; suspected PAD, suspected peripheral arterial disease—ABI less than 0.9. The dyslipidemia group included patients with total cholesterol > 5 mmol/L, LDL-C > 3 mmol/L, triglycerides > 1.7 mmol/L, and HDL-C < 1.0 mmol/L for men or <1.2 mmol/L for women, as well as those receiving statin therapy regardless of achieved lipid values. Reference intervals for uric acid: men 208.3–428.4 μmol/L; women 154.7–357 μmol/L.

**Table 6:** Medication use in patients who underwent CCTA grouped by CAD-RADS score.

	Groups by CAD-RADS Score				*p* Value
	Group 1 (*n* = 18)	Group 2 (*n* = 35)	Group 3 (*n* = 12)	Group 4 (*n* = 13)	
Statin, *n* (%)	4 (22.2)	10 (28.6)	6 (50.0)	7 (53.8)	*0.160*
ACE inhibitor/ARB, *n* (%)	8 (44.8) ^a^	11 (31.4) ^b^	9 (75.0) ^a,b^	9 (69.2) ^a,b^	*0.020*
Beta-blocker, *n* (%)	5 (27.8) ^c^	9 (25.7) ^d^	7 (58.3) ^c,d^	8 (61.5) ^c,d^	*0.041*
Calcium channel blocker, *n* (%)	4 (22.2)	2 (5.7) ^e^	4 (33.3)	5 (38.5) ^e^	*0.031*

ACE, angiotensin-converting enzyme; ARB, angiotensin II receptor blocker. Chi-square test for homogeneity was used. Results are presented as *n* (%). Superscript letters (a,b,c,d,e) indicate statistically significant differences between groups according to the Z-test with Bonferroni correction (*p* < 0.05).

**Table 7:** Lipid profile distribution in patient groups based on the presence of CAD.

	CAD Group (*n* = 71)	Non-CAD Group (*n* = 22)	*p* Value
Total cholesterol, mmol/L	5.9 (1.4)	5.4 (1.1)	*0.118*
LDL-C, mmol/L	3.9 (1.1)	3.3 (0.9)	*0.027*
HDL-C, mmol/L	1.5 (0.4)	1.6 (0.5)	*0.216*
Non-HDL-C, mmol/L	4.4 (1.3)	3.8 (1.1)	*0.038*
Triglycerides, mmol/L	1.3 (0.6)	1.1 (0.4)	*0.317*
ApoB, g/L	0.900 (0.268)	0.894 (0.164)	*0.951*
Lp(a), g/L	0.325 (0.370)	0.277 (0.269)	*0.475*
oxLDL, ng/mL	79.26 (29.01–257.56)	72.48 (31.30–139.05)	*0.190* *

Student’s *t*-test, data are presented as means (standard deviation). * Mann–Whitney test, data are presented as medians (minimum–maximum). LDL-C, low-density lipoprotein cholesterol; HDL, high-density lipoprotein cholesterol; Non-HDL-C, non-high-density lipoprotein cholesterol; ApoB, apolipoprotein B; Lp(a), lipoprotein (a); oxLDL, oxidized low-density lipoprotein. Reference intervals for ApoB: men 0.6–1.4 g/L; women 0.63–1.14 g/L; for Lp(a): 0.1–0.3 g/L.

**Table 8:** Lipid profile distribution in ICA patients stratified by Gensini score.

	Groups by Gensini Score			Group 4 (Patients Without CAD) (*n* = 7)	*p* Value
	Group 1 (*n* = 10)	Group 2 (*n* = 9)	Group 3 (*n* = 9)		
Total cholesterol, mmol/L	5.6 (3.0–8.1)	6.5 (4.5–8.1)	5.5 (3.6–8.1)	5.5 (4.0–7.4)	*0.550*
LDL-C, mmol/L	3.7 (1.4–5.2)	4.2 (2.8–5.5)	3.5 (1.9–6.4)	3.5 (2.3–5.3)	*0.799*
HDL-C, mmol/L	1.4 (1.1–2.8)	2.0 (0.8–2.4)	1.3 (1.0–1.8)	1.3 (1.0–1.5)	*0.467*
Non-HDL-C, mmol/L	4.1 (1.6–6.0)	4.5 (3.2–5.9)	4.0 (2.3–7.0)	4.2 (3.0–6.2)	*0.823*
Triglycerides, mmol/L	1.4 (0.4–1.6)	0.9 (0.8–1.9)	1.5 (0.6–2.6)	1.1 (0.9–2.7)	*0.437*
ApoB, g/L	0.851 (0.175–1.355)	0.836 (0.453–0.990)	0.902 (0.210–1.613)	0.801 (0.659–1.328)	*0.966*
Lp(a), g/L	0.212 (0.016–0.751)	0.144 (0.034–1.381)	0.456 (0.035–1.374)	0.245 (0.055–1.049)	*0.699*
oxLDL, ng/mL	69.25 (33.33–89.86) ^b^	97.19 (56.60–148.20) ^a,b^	95.81 (55.45–257.56) ^c^	52.58 (31.30–117.98) ^a,c^	*0.031* *0.026* ^a,b^ *0.049* ^c^

Kruskal–Wallis test, data are presented as medians (minimum–maximum). Superscript letters (a,b,c) indicate statistically significant differences between groups based on pairwise post hoc comparisons with Bonferroni correction following the Kruskal–Wallis test (*p* < 0.05). LDL-C, low-density lipoprotein cholesterol; HDL, high-density lipoprotein cholesterol; Non-HDL-C, non-high-density lipoprotein cholesterol; ApoB, apolipoprotein B; Lp(a), lipoprotein (a); oxLDL, oxidized low-density lipoprotein. Group 1 consisted of patients with a Gensini score between 0 and 10; Group 2 consisted of patients with a Gensini score between 11 and 30; Group 3 consisted of patients with a Gensini score above 31. Reference intervals for ApoB: men 0.6–1.4 g/L; women 0.63–1.14 g/L; for Lp(a): 0.1–0.3 g/L.

**Table 9:** Lipid profile distribution in CCTA patient groups according to CAD-RADS score.

	Groups by CAD-RADS Score				*p* Value
	Group 1 (*n* = 18)	Group 2 (*n* = 35)	Group 3 (*n* = 12)	Group 4 (*n* = 13)	
Total cholesterol, mmol/L	5.3 (3.7–6.9)	5.6 (3.5–8.0)	6.7 (3.0–8.5)	5.4 (3.6–9.7)	*0.061*
LDL-C, mmol/L	3.2 (2.0–4.6) ^a^	3.7 (2.0–5.5)	4.6 (1.4–5.6) ^a^	3.2 (1.9–6.7)	*0.032* *0.021* ^a^
HDL-C, mmol/L	1.6 (0.8–2.7)	1.4 (0.9–2.3)	1.5 (1.0–2.8)	1.4 (0.8–2.1)	*0.105*
Non-HDL-C, mmol/L	3.8 (2.1–5.2) ^b^	4.2 (2.2–6.6)	5.2 (1.6–6.6) ^b^	3.9 (2.3–8.4)	*0.046* *0.035* ^b^
Triglycerides, mmol/L	1.0 (0.5–2.1)	1.1 (0.6–2.7)	1.3 (0.4–2.6)	1.4 (0.8–3.7)	*0.330*
ApoB, g/L	0.910 (0.553–1.235)	0.871 (0.559–1.305)	0.980 (0.175–1.424)	0.943 (0.652–1.613)	*0.525*
Lp(a), g/L	0.178 (0.032–1.007)	0.167 (0.019–0.897)	0.116 (0.035–1.375)	0.197 (0.016–1.381)	*0.838*
oxLDL, ng/mL	80.63 (34.12–139.05)	69.88 (29.01–180.48)	79.79 (48.35–134.70)	85.89 (56.60–147.48)	*0.210*

Kruskal–Wallis test, data are presented as medians (minimum–maximum). Superscript letters (a,b) indicate statistically significant differences between groups based on pairwise post hoc comparisons following the Kruskal–Wallis test (*p* < 0.05). LDL-C, low-density lipoprotein cholesterol; HDL, high-density lipoprotein cholesterol; Non-HDL-C, non-high-density lipoprotein cholesterol; ApoB, apolipoprotein B; Lp(a), lipoprotein (a); oxLDL, oxidized low-density lipoprotein. Reference intervals for ApoB: men 0.6–1.4 g/L; women 0.63–1.14 g/L; for Lp(a): 0.1–0.3 g/L.

**Table 10:** Comparison of vimentin and endocan levels in patients with and without CAD.

	CAD Group (*n* = 71)	Non-CAD Group (*n* = 22)	*p* Value
Vimentin, ng/mL	186.9 (7.8–2081.0)	72.4 (7.8–399.0)	*0.012*
Endocan, pg/mL	187.2 (47.1–2382.1)	213.6 (84.5–2173.5)	*0.525*

Mann–Whitney test, data are presented as medians (minimum–maximum).

**Table 11:** Comparison of vimentin and endocan levels in ICA patients stratified by Gensini score.

	Groups by Gensini Score			Group 4 (Patients Without CAD) (*n* = 7)	*p* Value
	Group 1 (*n* = 10)	Group 2 (*n* = 9)	Group 3 (*n* = 9)		
Vimentin, ng/mL	118.7 (7.8–950.0)	379.0 (76.2–849.0)	425.9 (70.2–1485.0)	95.4 (7.8–1209.0)	*0.105*
Endocan, pg/mL	161.7 (47.1–275.4)	177.6 (113.0–452.5)	189.3 (88.9–556.5)	235.7 (118.3–330.6)	*0.555*

Kruskal–Wallis test, data are presented as medians (minimum–maximum).

**Table 12:** Comparison of vimentin and endocan levels in CCTA patients grouped by CAD-RADS score.

	Groups by CAD-RADS Score				*p* Value
	Group 1 (*n* = 18)	Group 2 (*n* = 35)	Group 3 (*n* = 12)	Group 4 (*n* = 13)	
Vimentin, ng/mL	70.8 (7.8–399.0)	169.5 (7.8–2081.0)	216.0 (7.8–849.0)	314.5 (70.2–1485.0)	*0.077*
Endocan, pg/mL	171.3 (84.5–2173.5)	305.9 (48.4–2382.1)	192.4 (54.9–1093.7)	162.4 (88.9–266.0)	*0.343*

Kruskal–Wallis test, data are presented as medians (minimum–maximum).

**Table 13:** Comparison of TSP-1 and TSP-2 levels between patients with and without CAD.

	CAD Group (*n* = 71)	Non-CAD Group (*n* = 22)	*p* Value
TSP-1 ng/mL	2.84 (0.59–11.53)	5.59 (1.21–8.00)	*0.209*
TSP-2 ng/mL	3.8 (0.5–30.0)	3.4 (0.5–30.0)	*0.811*

Mann–Whitney test, data are presented as medians (minimum–maximum). TSP-1, thrombospondin-1; TSP-2, thrombospondin-2.

**Table 14:** Comparison of TSP-1 and TSP-2 levels in ICA patients stratified by Gensini score.

	Groups by Gensini Score			Group 4 (Patients Without CAD) (*n* = 7)	*p* Value
	Group 1 (*n* = 10)	Group 2 (*n* = 9)	Group 3 (*n* = 9)		
TSP-1 ng/mL	3.65 (1.87–8.00)	2.67 (1.51–8.00)	3.18 (1.15–8.00)	4.18 (1.53–8.00)	*0.805*
TSP-2 ng/mL	5.0 (1.2–30.0)	3.4 (0.5–30.0)	10.8 (3.3–30.0)	3.81 (1.72–30.0)	*0.397*

Kruskal–Wallis test, data are presented as medians (minimum–maximum values). TSP-1, thrombospondin-1; TSP-2, thrombospondin-2.

**Table 15:** Comparison of TSP-1 and TSP-2 levels among CCTA patients stratified by CAD-RADS score.

	Groups by CAD-RADS Score				*p* Value
	Group 1 (*n* = 18)	Group 2 (*n* = 35)	Group 3 (*n* = 12)	Group 4 (*n* = 13)	
TSP-1 ng/mL	5.97 (1.21–8.00)	3.41 (1.12–11.53)	2.44 (0.59–8.00)	3.56 (1.15–8.00)	*0.468*
TSP-2 ng/mL	3.4 (0.5–30.0)	3.5 (0.9–30.0)	3.8 (0.5–30.0)	6.2 (0.5–30.0)	*0.914*

Kruskal–Wallis test, data are presented as medians (minimum–maximum). TSP-1, thrombospondin-1; TSP-2, thrombospondin-2.

**Table 16:** Associations between oxLDL levels and selected clinical parameters (Spearman correlation).

Variables	OxLDL Levels		
	Correlation Coefficient (r)	95% Confidence Interval	*p* Value
Systolic BP (mmHg)	0.304	[0.095; 0.488]	*0.004*
ABI-R	−0.322	[−0.509; −0.107]	*0.003*
ABI-L	−0.313	[−0.501; −0.096]	*0.004*

BP, blood pressure; ABI-R, right-side ankle–brachial index; ABI-L, left-side ankle–brachial index; r, Spearman’s correlation coefficient. A *p* value less than 0.05 was considered statistically significant.

**Table 17:** Spearman correlations between serum oxLDL levels and indicators of atherosclerosis severity.

Variables	OxLDL Levels		
	Correlation Coefficient (r)	95% Confidence Interval	*p* Value
Gensini score	0.455	[0.128; 0.693]	*0.006*
Need for PCI	0.318	[0.110; 0.499]	*0.003*
No. of atherosclerotic CA segments (ICA)	0.469	[0.146; 0.702]	*0.005*
No. of atherosclerotic CAs (ICA)	0.479	[0.157; 0.708]	*0.004*
RCx significant stenosis (ICA)	0.323	[0.195; 0.727]	*0.005*

OxLDL, oxidized low-density lipoproteins; Gensini score, a quantitative method for assessing the severity of coronary artery disease, based on the degree and anatomical location of arterial narrowing; need for PCI, presence of significant coronary artery disease requiring percutaneous coronary intervention; No. of atherosclerotic CA segments (ICA), number of coronary artery segments with atherosclerotic lesions detected by invasive coronary angiography (ICA); No. of atherosclerotic CAs (ICA), number of coronary arteries affected by atherosclerosis as detected by ICA; RCx significant stenosis (ICA), ramus circumflexus with ≥70% stenosis detected by ICA; ICA, invasive coronary angiography; r, Spearman’s correlation coefficient. A *p* value less than 0.05 was considered statistically significant.

**Table 18:** Spearman correlations between vimentin levels and indicators of atherosclerosis severity.

Variables	Vimentin Levels		
	Correlation Coefficient (r)	95% Confidence Interval	*p* Value
Gensini score	0.480	[0.148; 0.715]	*0.005*
SIS	0.349	[0.115; 0.546]	*0.003*
SSS	0.320	[0.079; 0.525]	*0.008*
No. of atherosclerotic CAs (CCTA)	0.322	[0.085; 0.524]	*0.007*
Need for PCI	0.324	[0.110; 0.509]	*0.003*
No. of atherosclerotic CA segments (ICA)	0.515	[0.192; 0.737]	*0.003*
No. of atherosclerotic CAs (ICA)	0.384	[0.030; 0.652]	*0.030*
CAD-RADS score	0.331	[0.095; 0.532]	*0.005*

Gensini score, a quantitative method for assessing the severity of coronary artery disease, based on the degree and anatomical location of arterial narrowing; SIS, segment involvement score; SSS, segment stenosis score; No. of atherosclerotic CAs (CCTA), number of coronary arteries affected by atherosclerosis, as detected by CCTA; CCTA, coronary computed tomography angiography; need for PCI, presence of significant coronary artery disease requiring percutaneous coronary intervention; No. of atherosclerotic CA segments (ICA), number of coronary artery segments with atherosclerotic lesions, as detected by invasive coronary angiography (ICA); No. of atherosclerotic CAs (ICA), number of coronary arteries affected by atherosclerosis, as detected by ICA; ICA, invasive coronary angiography; CAD-RADS, coronary artery disease-reporting and data system—a classification system for grading coronary stenosis severity based on CCTA findings; r, Spearman’s correlation coefficient. A *p* value less than 0.05 was considered statistically significant.

## Data Availability

The original contributions presented in this study are included in the article. Further inquiries can be directed to the corresponding author.
